# Tris(ethyl­enediamine-κ^2^ *N*,*N*′)cadmium hexa­fluoridogermanate

**DOI:** 10.1107/S160053681200983X

**Published:** 2012-03-10

**Authors:** Guo-Ming Wang, Zeng-Xin Li, Pei Wang

**Affiliations:** a Teachers College, College of Chemistry, Chemical Engineering and Environment, Qingdao University, Shandong 266071, People’s Republic of China; b Teachers College, Qingdao University, Shandong 266071, People’s Republic of China; c College of Chemistry, Chemical Engineering and Environment, Qingdao University, Shandong 266071, People’s Republic of China

## Abstract

In the title compound, [Cd(C_2_H_8_N_2_)_3_](GeF_6_), the Cd^II^ atom, lying on a 32 symmetry site, is coordinated by six N atoms from three ethyl­enediamine (en) ligands in a distorted octa­hedral geometry. The Ge atom also lies on a 32 symmetry site and is coordinated by six F atoms. The en ligand has a twofold rotation axis passing through the mid-point of the C—C bond. The F atom is disordered over two sites with equal occupancy factors. In the crystal, the [Cd(en)_3_]^2+^ cations and [GeF_6_]^2−^ anions are connected through N—H⋯F hydrogen bonds, forming a three-dimensional supra­molecular network.

## Related literature

For background to the structures and applications of microporous materials, see: Cheetham *et al.* (1999 ▶); Jiang *et al.* (2010 ▶); Liang *et al.* (2006 ▶); Yu & Xu (2003 ▶); Zou *et al.* (2005 ▶). For related fluorides, see: Brauer *et al.* (1980 ▶, 1986 ▶); Dadachov *et al.* (2001 ▶); Lukevics *et al.* (1997 ▶); Tang *et al.* (2001*a* ▶,*b* ▶,*c* ▶,*d* ▶,*e* ▶,*f* ▶); Wang *et al.* (2004 ▶); Wang & Wang (2011 ▶); Zhang *et al.* (2003 ▶). For related structures containing chiral metal complexes, see: Stalder & Wilkinson (1997 ▶); Wang *et al.* (2003 ▶); Yu *et al.* (2001 ▶).
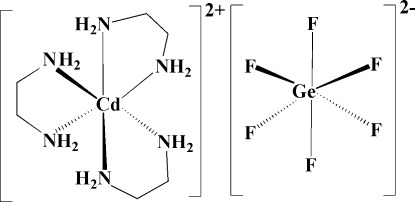


## Experimental

### 

#### Crystal data

[Cd(C_2_H_8_N_2_)_3_](GeF_6_)
*M* *_r_* = 479.33
Trigonal, 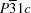

*a* = 9.5422 (3) Å
*c* = 9.9977 (5) Å
*V* = 788.37 (7) Å^3^
*Z* = 2
Mo *K*α radiation
μ = 3.32 mm^−1^
*T* = 293 K
0.20 × 0.18 × 0.12 mm

#### Data collection

Bruker APEX CCD diffractometer
Absorption correction: multi-scan (*SADABS*; Sheldrick, 1996 ▶) *T*_min_ = 0.557, *T*_max_ = 0.692
7348 measured reflections
549 independent reflections
496 reflections with *I* > 2σ(*I*)
*R*_int_ = 0.038

#### Refinement

*R*[*F* ^2^ > 2σ(*F* ^2^)] = 0.024
*wR*(*F* ^2^) = 0.038
*S* = 1.16
549 reflections
42 parameters
12 restraints
H-atom parameters constrained
Δρ_max_ = 0.23 e Å^−3^
Δρ_min_ = −0.23 e Å^−3^

### 

Data collection: *SMART* (Bruker, 2007 ▶); cell refinement: *SAINT* (Bruker, 2007 ▶); data reduction: *SAINT*; program(s) used to solve structure: *SHELXS97* (Sheldrick, 2008 ▶); program(s) used to refine structure: *SHELXL97* (Sheldrick, 2008 ▶); molecular graphics: *DIAMOND* (Brandenburg, 1999 ▶); software used to prepare material for publication: *SHELXTL* (Sheldrick, 2008 ▶).

## Supplementary Material

Crystal structure: contains datablock(s) global, I. DOI: 10.1107/S160053681200983X/hy2520sup1.cif

Structure factors: contains datablock(s) I. DOI: 10.1107/S160053681200983X/hy2520Isup2.hkl

Additional supplementary materials:  crystallographic information; 3D view; checkCIF report

## Figures and Tables

**Table 1:** Hydrogen-bond geometry (Å, °)

*D*—H⋯*A*	*D*—H	H⋯*A*	*D*⋯*A*	*D*—H⋯*A*
N1—H1*C*⋯F1^i^	0.90	2.28	3.135 (11)	158
N1—H1*C*⋯F1′^i^	0.90	2.06	2.959 (11)	173
N1—H1*D*⋯F1	0.90	1.94	2.831 (11)	172
N1—H1*D*⋯F1′	0.90	2.16	3.005 (11)	156

Symmetry code: (i) 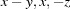.

**Fig. 1.**
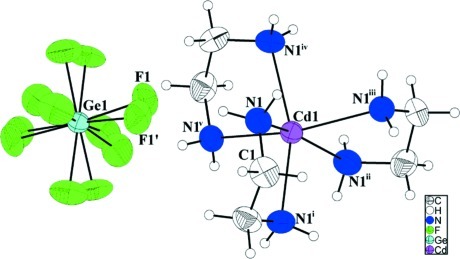
The molecular structure of (I). Displacement ellipsoids are drawn at the 50% probability level. [Symmetry codes: (i) -x + y, y, 1/2 - z; (ii) x, 1 + x - y, 1/2 - z; (iii) -x + y, 1 - x, z; (iv) 1 - y, 1 + x - y, z; (v) 1 - y, 1 - x, 1/2 - z.]

**Fig. 2.**
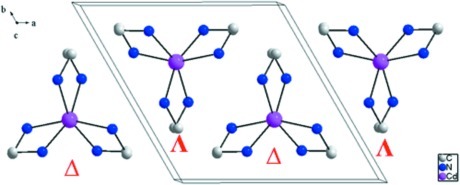
The arrangement of the chiral [Cd(en)3]2+ complexes along the a axis.

**Fig. 3.**
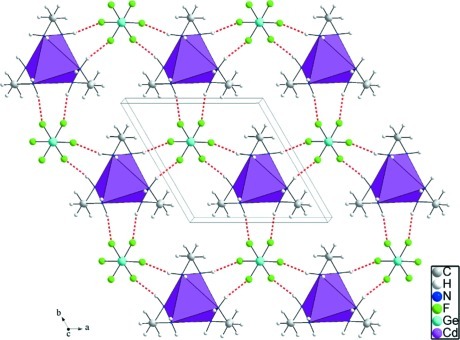
View of the hydrogen-bonded layer from the [Cd(en)3]2+ and [GeF6]2- ions.

**Fig. 4.**
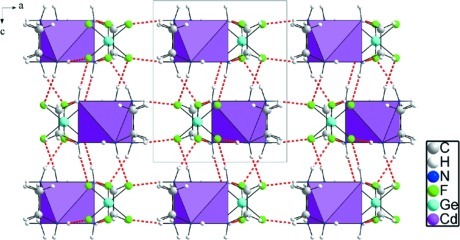
The expansion of adjacent layers into a three-dimensional hydrogen-bonded network.

## supplementary crystallographic information

### Comment

In recent years, there has been much interest in the design and synthesis of crystalline microporous materials because of their rich structural chemistry and potential applications in catalysis, ion-exchange and separation (Cheetham *et al.*, 1999; Jiang *et al.*, 2010; Liang *et al.*, 2006; Yu & Xu, 2003; Zou *et al.*, 2005). In addition to the most notable zeolites, many non-aluminosilicate-based microporous systems, such as metal phosphates, germanates, borates, *etc*. have been extensively investigated. In contrast, the progress in the field of fluorides has been limited, though some fluoroaluminates (Tang *et al.*, 2001*c*,e), fluorosilicate (Tang *et al.*, 2001*f*), fluorotitanates (Dadachov *et al.*, 2001; Tang *et al.*, 2001*a*,b,d) and fluorogermanates (Brauer *et al.*, 1980,1986; Lukevics *et al.*, 1997; Wang *et al.*, 2004; Wang & Wang, 2011; Zhang *et al.*, 2003) have been reported. The main purpose of our work is to prepare microporous germanates templated by transition-metal complexes. Unexpectedly, the title compound, (I), was obtained, which is a new fluorogermanate templated by [Cd(en)_3_]^2+^ cations (en = ethylenediamine).

The crystal structure of (I) consists of discrete [Cd(en)_3_]^2+^ cations and [GeF_6_]^2-^ anions (Fig. 1). Both of the cation and anion lie on 32 symmetry sites. In the [GeF_6_]^2-^ anion, the Ge atom is six-coordinated in a distorted octahedral geometry by six symmetry-related F atoms. The Ge—F bond distances are 1.812 (9) and 1.746 (9) Å, similar to the distances observed in inorganic complex K_2_GeF_6_ (Ge—F 1.77 Å) and in other fluorogermanates. In the [Cd(en)_3_]^2+^ cation, the Cd^II^ atom is bonded to six amine N aoms from three symmetry-related en ligands. The Cd—N bond distance is 2.370 (2) Å, comparable with those found in other related compounds. Interestingly, the [Cd(en)_3_]^2+^ complex generated *in situ* is chiral, and the enantiomers are alternately arranged along the *a* axis (Fig. 2). It is worthy to note that the rigid octahedrally coordinated metal amine complex with chiral features is particularly rare and usually characterized as Co and Ir complexes, such as [Co(en)_3_]^3+^, [Co(tn)_3_]^3+^ (tn = 1,3-diaminopropane), [Co(dien)_2_]^3+^ (dien = diethylenetriamine), [Ir(en)_3_]^3+^, etc (Stalder & Wilkinson, 1997; Wang *et al.*, 2003; Yu *et al.*, 2001). Each [Cd(en)_3_]^2+^ cation is linked to three neighboring [GeF_6_]^2-^ anions through N1—H1D···F1 hydrogen bonds (Table 1), generating a hydrogen-bonded layer along [001] (Fig. 3). Adjacent layers are further connected with each other through N1—H1C···F1 hydrogen bonds (Fig. 4), giving rise to a three-dimensional supramolecular network .

### Experimental

The title compound was obtained by hydrothermal methods. Typically, a mixture of GeO_2_ (0.104 g, 1 mmol), CdCO_3_ (0.174 g, 1 mmol), en (1.34 ml), pyridine (2.50 ml), hydrofluoric acid (40%, 0.20 ml) and H_2_O (1.00 ml) in a molar ratio of 1:1:20:31:10:56 was sealed in a 25 ml Teflon-lined steel autoclave and heated under autogenous pressure at 443 K for 7 days. The block crystals obtained were recovered by filtration, washed with distilled water and dried in air.

### Refinement

Atom F1 was refined as disordered over two positions, each with 50% site occupancy. All H atoms were positioned geometrically and refined as riding atoms, with C—H = 0.97 and N—H = 0.90 Å and with *U*_iso_(H) = 1.2*U*_eq_(C, N).

### Crystal data

**Table:**

[Cd(C_2_H_8_N_2_)_3_](GeF_6_)	*D*_x_ = 2.019 Mg m^−^^3^
*M**_r_* = 479.33	Mo *K*α radiation, λ = 0.71073 Å
Trigonal, *P*31*c*	Cell parameters from 549 reflections
Hall symbol: -P 3 2c	θ = 4.1–26.5°
*a* = 9.5422 (3) Å	µ = 3.32 mm^−^^1^
*c* = 9.9977 (5) Å	*T* = 293 K
*V* = 788.37 (7) Å^3^	Block, colorless
*Z* = 2	0.20 × 0.18 × 0.12 mm
*F*(000) = 472	

### Data collection

**Table:**

Bruker APEX CCD diffractometer	549 independent reflections
Radiation source: fine-focus sealed tube	496 reflections with *I* > 2σa(*I*)
Graphite monochromator	*R*_int_ = 0.038
φ and ω scans	θ_max_ = 26.5°, θ_min_ = 4.1°
Absorption correction: multi-scan (*SADABS*; Sheldrick, 1996)	*h* = −11→11
*T*_min_ = 0.557, *T*_max_ = 0.692	*k* = −11→11
7348 measured reflections	*l* = −12→12

### Refinement

**Table:**

Refinement on *F*^2^	Secondary atom site location: difference Fourier map
Least-squares matrix: full	Hydrogen site location: inferred from neighbouring sites
*R*[*F*^2^ > 2σ(*F*^2^)] = 0.024	H-atom parameters constrained
*wR*(*F*^2^) = 0.038	*w* = 1/[σ^2^(*F*_o_^2^) + (0.0048*P*)^2^ + 0.9629*P*] where *P* = (*F*_o_^2^ + 2*F*_c_^2^)/3
*S* = 1.16	(Δ/σ)_max_ < 0.001
549 reflections	Δρ_max_ = 0.23 e Å^−^^3^
42 parameters	Δρ_min_ = −0.23 e Å^−^^3^
12 restraints	Extinction correction: *SHELXL97* (Sheldrick, 2008), Fc^*^=kFc[1+0.001xFc^2^λ^3^/sin(2θ)]^-1/4^
Primary atom site location: structure-invariant direct methods	Extinction coefficient: 0.0045 (3)

### Special details

**Table:**

Geometry. All e.s.d.'s (except the e.s.d. in the dihedral angle between two l.s. planes) are estimated using the full covariance matrix. The cell e.s.d.'s are taken into account individually in the estimation of e.s.d.'s in distances, angles and torsion angles; correlations between e.s.d.'s in cell parameters are only used when they are defined by crystal symmetry. An approximate (isotropic) treatment of cell e.s.d.'s is used for estimating e.s.d.'s involving l.s. planes.
Refinement. Refinement of *F*^2^ against ALL reflections. The weighted *R*-factor *wR* and goodness of fit *S* are based on *F*^2^, conventional *R*-factors *R* are based on *F*, with *F* set to zero for negative *F*^2^. The threshold expression of *F*^2^ > σ(*F*^2^) is used only for calculating *R*-factors(gt) *etc*. and is not relevant to the choice of reflections for refinement. *R*-factors based on *F*^2^ are statistically about twice as large as those based on *F*, and *R*- factors based on ALL data will be even larger.

### Fractional atomic coordinates and isotropic or equivalent isotropic displacement parameters (Å^2^)

**Table:**

	*x*	*y*	*z*	*U*_iso_*/*U*_eq_	Occ. (<1)
Cd1	0.3333	0.6667	0.2500	0.03249 (17)	
Ge1	0.6667	0.3333	0.2500	0.02960 (19)	
N1	0.2829 (3)	0.4387 (3)	0.1196 (2)	0.0444 (6)	
H1C	0.2637	0.4538	0.0341	0.053*	
H1D	0.3698	0.4254	0.1212	0.053*	
C1	0.1425 (4)	0.2956 (4)	0.1743 (3)	0.0504 (8)	
H1A	0.1381	0.1989	0.1388	0.060*	
H1B	0.0443	0.2949	0.1479	0.060*	
F1	0.5391 (11)	0.3708 (8)	0.1387 (10)	0.065 (2)	0.50
F1'	0.5004 (11)	0.2974 (8)	0.1521 (10)	0.064 (2)	0.50

### Atomic displacement parameters (Å^2^)

**Table:**

	*U*^11^	*U*^22^	*U*^33^	*U*^12^	*U*^13^	*U*^23^
Cd1	0.0326 (2)	0.0326 (2)	0.0323 (3)	0.01630 (10)	0.000	0.000
Ge1	0.0312 (3)	0.0312 (3)	0.0264 (4)	0.01559 (13)	0.000	0.000
N1	0.0559 (17)	0.0475 (16)	0.0364 (13)	0.0309 (14)	−0.0008 (12)	−0.0037 (12)
C1	0.056 (2)	0.0397 (18)	0.0518 (18)	0.0214 (16)	−0.0092 (16)	−0.0112 (14)
F1	0.074 (5)	0.092 (5)	0.051 (3)	0.058 (4)	−0.012 (3)	0.001 (4)
F1'	0.049 (4)	0.098 (5)	0.049 (3)	0.040 (4)	−0.021 (3)	−0.010 (4)

### Geometric parameters (Å, º)

**Table:**

Cd1—N1^i^	2.370 (2)	Ge1—F1^vi^	1.812 (9)
Cd1—N1^ii^	2.370 (2)	Ge1—F1^viii^	1.812 (9)
Cd1—N1^iii^	2.370 (2)	Ge1—F1^ix^	1.812 (9)
Cd1—N1	2.370 (2)	Ge1—F1^v^	1.812 (9)
Cd1—N1^iv^	2.370 (2)	Ge1—F1	1.812 (9)
Cd1—N1^v^	2.370 (2)	N1—C1	1.459 (4)
Ge1—F1'^vi^	1.746 (9)	N1—H1C	0.9000
Ge1—F1'^vii^	1.746 (9)	N1—H1D	0.9000
Ge1—F1'^viii^	1.746 (9)	C1—C1^iii^	1.518 (6)
Ge1—F1'^v^	1.746 (9)	C1—H1A	0.9700
Ge1—F1'^ix^	1.746 (9)	C1—H1B	0.9700
Ge1—F1'	1.746 (9)	F1—F1'	0.621 (10)
Ge1—F1^vii^	1.812 (9)		
			
N1^i^—Cd1—N1^ii^	74.72 (12)	F1'^ix^—Ge1—F1^viii^	176.1 (7)
N1^i^—Cd1—N1^iii^	92.62 (8)	F1'—Ge1—F1^viii^	91.4 (3)
N1^ii^—Cd1—N1^iii^	103.54 (12)	F1^vii^—Ge1—F1^viii^	86.2 (5)
N1^i^—Cd1—N1	159.72 (12)	F1^vi^—Ge1—F1^viii^	82.4 (6)
N1^ii^—Cd1—N1	92.62 (8)	F1'^vi^—Ge1—F1^ix^	73.7 (3)
N1^iii^—Cd1—N1	74.72 (12)	F1'^vii^—Ge1—F1^ix^	90.6 (2)
N1^i^—Cd1—N1^iv^	103.54 (12)	F1'^viii^—Ge1—F1^ix^	176.1 (7)
N1^ii^—Cd1—N1^iv^	92.62 (8)	F1'^v^—Ge1—F1^ix^	91.4 (3)
N1^iii^—Cd1—N1^iv^	159.72 (12)	F1'—Ge1—F1^ix^	100.8 (2)
N1—Cd1—N1^iv^	92.62 (8)	F1^vii^—Ge1—F1^ix^	82.4 (6)
N1^i^—Cd1—N1^v^	92.62 (8)	F1^vi^—Ge1—F1^ix^	86.2 (5)
N1^ii^—Cd1—N1^v^	159.72 (12)	F1^viii^—Ge1—F1^ix^	160.3 (5)
N1^iii^—Cd1—N1^v^	92.62 (8)	F1'^vi^—Ge1—F1^v^	176.1 (7)
N1—Cd1—N1^v^	103.54 (12)	F1'^vii^—Ge1—F1^v^	100.8 (2)
N1^iv^—Cd1—N1^v^	74.72 (12)	F1'^viii^—Ge1—F1^v^	73.7 (3)
F1'^vi^—Ge1—F1'^vii^	76.2 (6)	F1'^ix^—Ge1—F1^v^	91.4 (3)
F1'^vi^—Ge1—F1'^viii^	103.8 (6)	F1'—Ge1—F1^v^	90.6 (2)
F1'^vii^—Ge1—F1'^viii^	91.7 (5)	F1^vii^—Ge1—F1^v^	86.2 (5)
F1'^vi^—Ge1—F1'^v^	160.4 (5)	F1^vi^—Ge1—F1^v^	160.3 (5)
F1'^vii^—Ge1—F1'^v^	91.7 (5)	F1^viii^—Ge1—F1^v^	86.2 (5)
F1'^viii^—Ge1—F1'^v^	91.7 (5)	F1^ix^—Ge1—F1^v^	108.8 (5)
F1'^vi^—Ge1—F1'^ix^	91.7 (5)	F1'^vi^—Ge1—F1	100.8 (2)
F1'^vii^—Ge1—F1'^ix^	103.8 (6)	F1'^vii^—Ge1—F1	176.1 (7)
F1'^viii^—Ge1—F1'^ix^	160.4 (5)	F1'^viii^—Ge1—F1	91.4 (3)
F1'^v^—Ge1—F1'^ix^	76.2 (6)	F1'^v^—Ge1—F1	90.6 (2)
F1'^vi^—Ge1—F1'	91.7 (5)	F1'^ix^—Ge1—F1	73.7 (3)
F1'^vii^—Ge1—F1'	160.4 (5)	F1^vii^—Ge1—F1	160.3 (5)
F1'^viii^—Ge1—F1'	76.2 (6)	F1^vi^—Ge1—F1	86.2 (5)
F1'^v^—Ge1—F1'	103.8 (6)	F1^viii^—Ge1—F1	108.8 (5)
F1'^ix^—Ge1—F1'	91.7 (5)	F1^ix^—Ge1—F1	86.2 (5)
F1'^vi^—Ge1—F1^vii^	91.4 (3)	F1^v^—Ge1—F1	82.4 (6)
F1'^viii^—Ge1—F1^vii^	100.8 (2)	C1—N1—Cd1	108.83 (17)
F1'^v^—Ge1—F1^vii^	73.7 (3)	C1—N1—H1C	109.9
F1'^ix^—Ge1—F1^vii^	90.6 (2)	Cd1—N1—H1C	109.9
F1'—Ge1—F1^vii^	176.1 (7)	C1—N1—H1D	109.9
F1'^vii^—Ge1—F1^vi^	91.4 (3)	Cd1—N1—H1D	109.9
F1'^viii^—Ge1—F1^vi^	90.6 (2)	H1C—N1—H1D	108.3
F1'^v^—Ge1—F1^vi^	176.1 (7)	N1—C1—C1^iii^	110.1 (2)
F1'^ix^—Ge1—F1^vi^	100.8 (2)	N1—C1—H1A	109.6
F1'—Ge1—F1^vi^	73.7 (3)	C1^iii^—C1—H1A	109.6
F1^vii^—Ge1—F1^vi^	108.8 (5)	N1—C1—H1B	109.6
F1'^vi^—Ge1—F1^viii^	90.6 (2)	C1^iii^—C1—H1B	109.6
F1'^vii^—Ge1—F1^viii^	73.7 (3)	H1A—C1—H1B	108.2
F1'^v^—Ge1—F1^viii^	100.8 (2)		

Symmetry codes: (i) *x*, *x*−*y*+1, −*z*+1/2; (ii) −*x*+*y*, −*x*+1, *z*; (iii) −*x*+*y*, *y*, −*z*+1/2; (iv) −*y*+1, *x*−*y*+1, *z*; (v) −*y*+1, −*x*+1, −*z*+1/2; (vi) −*y*+1, *x*−*y*, *z*; (vii) −*x*+*y*+1, *y*, −*z*+1/2; (viii) *x*, *x*−*y*, −*z*+1/2; (ix) −*x*+*y*+1, −*x*+1, *z*.

### Hydrogen-bond geometry (Å, º)

**Table:**

*D*—H···*A*	*D*—H	H···*A*	*D*···*A*	*D*—H···*A*
N1—H1*C*···F1^x^	0.90	2.28	3.135 (11)	158
N1—H1*C*···F1′^x^	0.90	2.06	2.959 (11)	173
N1—H1*D*···F1	0.90	1.94	2.831 (11)	172
N1—H1*D*···F1′	0.90	2.16	3.005 (11)	156

Symmetry code: (x) *x*−*y*, *x*, −*z*.
